# Supplementary material for: Reduction in Ventilation-Induced Diaphragmatic Mitochondrial Injury through Hypoxia-Inducible Factor 1α in a Murine Endotoxemia Model
Source: Int J Mol Sci. 2022 Jan 19;23(3):1083. doi: 10.3390/ijms23031083 (PMC8835058; doi:10.3390/ijms23031083)
Supplement: Supplementary file 1 [file ijms-23-01083-s001.zip › Table S1.pdf]

Table S1. Physiologic conditions at the beginning and end of ventilation.

	Nonventilated	Nonventilated LPS	V_T 10 ml/kg	V_T 10 ml/kg LPS	V_T 10 ml/kg LPS+HIF-1α
pH	7.42±0.06	7.39±0.03	7.38±0.05	7.37±0.06	7.37±0.05
PaO2 (mmHg)	98.3±0.4	92.1±0.4	86.3±0.4*	83.5±2.4*	86.3±2.4*
PaCO2 (mmHg)	39.0±0.3	39.7±0.3	36.8±1.2	38.4±1.2	37.8±1.5
MAP (mmHg)					
Start	85.7±1.3	83.8±0.4	85.1±1.3	83.3±2.1	84.9±2.3
End	85.2±0.3	81.2±0.3	77.5±2.4*	76.2±2.4*	78.6±2.5*
PIP (mmHg)					
Start			16.0±1.1	16.3±1.4	15.6±1.2
End			16.9±1.5	17.4±1.7	17.0±1.3

At the end of the study period, we obtained data of mean arterial pressure and arterial blood gases from the nonventilated control mice and mice ventilated at a tidal volume of 10 mL/kg for 8 h (n = 10 per group). The normovolemic statuses of mice were maintained by monitoring mean artery pressure. Data are presented as means ± SDs. * indicates that $P < 0.05$ when compared to the nonventilated control mice. HIF = hypoxia-inducible factor; LPS = lipopolysaccharide; MAP = mean arterial pressure; PIP = peak inspiratory pressure; V_T = tidal volume.
